# Draft Genome Sequence of Roseomonas aestuarii Strain JR1/69-1-13 Isolated from Nitrate- and Radionuclide-Contaminated Groundwater in Russia

**DOI:** 10.1128/genomeA.00583-18

**Published:** 2018-06-21

**Authors:** Denis S. Grouzdev, Tamara L. Babich, Tatiyana P. Tourova, Diyana S. Sokolova, Ruslan R. Abdullin, Andrey B. Poltaraus, Margarita A. Schevchenko, Stepan V. Toshchakov, Tamara N. Nazina

**Affiliations:** a Institute of Bioengineering, Research Center of Biotechnology, Russian Academy of Sciences, Moscow, Russian Federation; b Winogradsky Institute of Microbiology, Research Center of Biotechnology, Russian Academy of Sciences, Moscow, Russian Federation; c Engelhardt Institute of Molecular Biology, Russian Academy of Sciences, Moscow, Russian Federation; d Immanuel Kant Baltic Federal University, Kaliningrad, Russian Federation; e V. I. Vernadsky Institute of Geochemistry and Analytical Chemistry, Russian Academy of Sciences, Moscow, Russian Federation

## Abstract

The draft genome sequence of Roseomonas aestuarii strain JR1/69-1-13, an aerobic chemoorganotrophic bacterium isolated from nitrate- and radionuclide-contaminated groundwater in Russia, is presented here. The genome was annotated to elucidate the genomic basis for the strain’s adaptation to the environment and its resistance to nitrate, heavy metals, and metalloids.

## GENOME ANNOUNCEMENT

Members of the genus Roseomonas belong to the class Alphaproteobacteria and are pink, Gram-negative coccobacilli with an oxidative metabolism that have been isolated from clinical samples, drinking water, wetland freshwater, activated sludge, as well as estuarine habitat, air, and soil samples (1–3). R. aestuarii strain JR1/69-1-13 (=VKM B-3221) was isolated from a groundwater sample collected from an observation well (depth of 44 m) located at a distance of 3.2 km from Karachai Lake (Ozyorsk, South Urals, Russia; 55°38′N, 60°47′E), which was used for the storage of liquid radioactive waste. Strain JR1/69-1-13 is a Gram-negative, aerobic chemoorganotrophic bacterium that is motile in the early exponential phase and forms orange-pigmented colonies. The strain was able to grow at temperatures ranging from 13 to 42°C (optimum at 35°C) and at pH values ranging from 7.0 to 7.5 in the presence of 0 to 3% (wt/vol) NaCl (optimum at 0.5 to 1% [wt/vol] NaCl). The strain utilized sugars, amino acids, aromatic compounds, and organic acids. Under anaerobic conditions in the medium with glucose, strain JR1/69-1-13 reduced nitrate to nitrite. The 16S rRNA gene of strain JR1/69-1-13 had 100% similarity with the respective gene of R. aestuarii JC17^T^ (3), in spite of their physiological differences. The genome of the R. aestuarii type strain is presently not represented in the NCBI database.

Genomic DNA was isolated from the biomass by a phenol-chloroform method as described previously (4). Fragment libraries were prepared from 1 μg of genomic DNA with the NEBNext Ultra DNA library preparation kit (New England Biolabs, Ipswich, MA, USA) according to the manufacturer’s instructions, to obtain the mean library size of 700 bp. The genomic DNA library was sequenced using the Illumina MiSeq personal sequencing system (Illumina, Inc., San Diego, CA, USA), resulting in approximately 2,908,155 pairs of 150-bp reads. The reads were subjected to stringent quality trimming by CLC Genomics Workbench version 10.0 software (Qiagen, Germany). After filtering, 2,573,564 read pairs were used for the *de novo* assembly. The genome was assembled with the SPAdes version 3.10.0 genome assembler (5). After assembly, the contigs were filtered by length and coverage. Final assembly of the 5,195,849-bp total length consisted of 106 contigs with an *N*_50_ value of 245,408 bp. Average read coverage was 145×. The assembled sequence was submitted to the NCBI Prokaryotic Genome Annotation Pipeline (PGAP) (6) for annotation.

The draft genome sequence of strain JR1/69-1-13 revealed a genome size of 5,195,849 bp, with an average G+C content of 71.5%, which exceeded significantly the 66.2% value determined for the R. aestuarii type strain JC17 (3). The genome of strain JR1/69-1-13 contained 4,849 genes, of which 4,650 were coding DNA sequences, 52 were tRNAs, and 4 were noncoding RNAs. Numerous genes responsible for the utilization of a range of organic substrates (sugars, amino acids, organic acids, and chloroaromatic compounds), as well as for heavy metal tolerance and detoxification, were identified. We present here the draft genome sequence of R. aestuarii strain JR1/69-1-13 (=VKM B-3221) with the hope that these data will facilitate a deeper understanding of the molecular mechanisms of its resistance to nitrate, heavy metals, and metalloids and expand our knowledge of the physiology of members of the genus Roseomonas.

### Accession number(s).

This whole-genome shotgun project has been deposited at DDBJ/ENA/GenBank under the accession no. PDOA00000000. The version described in this paper is the first version, PDOA01000000.
